# Using neurocognitive models to optimise the treatment of depression

**DOI:** 10.1192/j.eurpsy.2023.40

**Published:** 2023-07-19

**Authors:** S. Murphy

**Affiliations:** Psychiatry, University of Oxford, Oxford, United Kingdom

## Abstract

**Abstract:**

Conventional antidepressants, such as SSRIs, are an effective treatment for many patients with depression. However, for a significant proportion of patients SSRIs either lack efficacy or are poorly tolerated. Even when SSRIs are effective in treating mood symptoms, there are often residual symptoms that are not well treated, including cognitive impairment and anhedonia. The development of novel treatment for depression is particularly challenging given the limited predictive validity of animal models. Human neurocognitive models of antidepressant action can help to bridge the translational gap and allow rapid investigation of novel compounds in healthy volunteers and people with depression. In this talk, using the 5-HT_4_ receptor as an example of a novel target of interest, I will outline how these objective neurocognitive models can be used as a translational tool to understand antidepressant treatment mechanisms, guide treatment selection and test novel putative antidepressants early in development.

**Disclosure of Interest:**

S. Murphy Grant / Research support from: Zogenix, UCB, Janssen, Consultant of: Zogenix, Sumitomo Danippon, Janssen, UCB, Speakers bureau of: Zogenix

